# Improvement of Steroid-Dependent Encephalopathy Clinically Diagnosed as Paraneoplastic Neurological Syndrome in Onconeural Antibody-Negative Sigmoid Colon Cancer after Laparoscopic Tumor Resection

**DOI:** 10.70352/scrj.cr.25-0805

**Published:** 2026-03-05

**Authors:** Susumu Inamoto, Shota Tsukasaki, Tomoaki Okada, Akinari Nomura, Yoshiharu Sakai

**Affiliations:** 1 Department of Gastroenterological Surgery, Japanese Red Cross Osaka Hospital, Osaka, Osaka, Japan; 2 Department of Surgery, Graduate School of Medicine, Kyoto University, Kyoto, Kyoto, Japan

**Keywords:** paraneoplastic neurological syndrome, impaired consciousness, onconeural autoantibody, colorectal cancer, prednisolone

## Abstract

**INTRODUCTION:**

Paraneoplastic neurological syndrome (PNS) is a rare complication of malignancy caused by immune-mediated mechanisms. Although characteristic onconeural antibodies are useful for the diagnosis of PNS, 30%–40% of cases are seronegative, which makes recognition difficult. We present a case of sigmoid colon cancer complicated by onconeural autoantibody-negative PNS which caused impaired consciousness. The patient’s symptoms recurred with steroid tapering but improved after resection of the primary tumor and radiofrequency ablation (RFA) of the metastasis. This case highlights the importance of considering PNS, even when onconeural antibodies are negative.

**CASE PRESENTATION:**

A 79-year-old man was referred for evaluation of sigmoid colon cancer detected during the investigation of bloody stools. He had been receiving 20 mg of prednisolone (PSL) for suspected arthritis and cellulitis and was later diagnosed with gout. After tapering and discontinuation of PSL, the patient developed recurrent arthritis, impaired consciousness with somnolence, and hypoxemia. Cardiac, infectious, cerebrovascular, and metabolic causes were excluded, raising the suspicion of PNS despite negative onconeural antibody testing. The patient’s symptoms improved with 40 mg of PSL but worsened when the dose was reduced. An iliopsoas abscess and disseminated intravascular coagulation were identified and treated with antibiotics and PSL. However, balancing the risk of infection against recurrent neurological decline was difficult. For this reason, the patient underwent a laparoscopic Hartmann procedure. Postoperatively, PSL was tapered to 2.5 mg; however, impaired consciousness recurred with evidence of early liver metastasis. RFA was then performed, leading to the resolution of symptoms.

**CONCLUSIONS:**

This case demonstrates an onconeural antibody-negative PNS associated with sigmoid colon cancer. Neurological symptoms emerged in association with both the primary tumor and subsequent liver metastasis and improved after treatment of each lesion, with milder symptoms observed at metastatic recurrence, consistent with a smaller tumor burden. Clinicians should consider PNS even in seronegative cases and emphasize tumor-directed therapy when immunosuppression is constrained by infection risk.

## Abbreviations

DIC
: disseminated intravascular coagulation
PNS
: paraneoplastic neurological syndrome
PSL
: prednisolone

## INTRODUCTION

Paraneoplastic neurological syndrome (PNS) is a diverse group of tumor-related neurological disorders thought to arise from immunological mechanisms that are distinct from typical tumor progression-related neurological disorders, such as direct tumor invasion and metastasis, nutritional disorders, metabolic disorders, coagulation disorders, side effects of chemotherapy and radiation therapy, and immunosuppression. PNS is classified into several clinical subtypes based on the main symptoms. In many cases, characteristic onconeural antibodies associated with each subtype are detected in the serum and cerebrospinal fluid (CSF) of patients, and tumors closely associated with each subtype have also been found. Neurological symptoms generally progress sub-acutely, resulting in severe physical disability. In approximately 80% of cases, the onset of neurological symptoms and antibody detection precede tumor detection by several months to years.^1)^ Antibody detection has gained attention as a marker for the diagnosis of PNS and early detection of tumors. Including PNS in the differential diagnosis based on patient symptoms may enable the early detection of potential tumors, making PNS clinically important.

However, PNS is not a syndrome caused by endocrine secretions from tumors but rather a syndrome resulting from the cross-reaction of onconeural antibodies against tumors. As a result, successful treatment of the tumor does not necessarily result in improvement of neurological findings. Furthermore, 30%–40% of PNS cases are onconeural antibody-negative, which makes determining a diagnosis based on the presence or absence of onconeural antibodies alone difficult.^2)^

Colorectal cancer is the third most common cancer worldwide, accounting for approximately 10% of all cancer cases and is the second leading cause of cancer-related deaths. The incidence and mortality rates for colorectal cancer tend to be higher in Europe, Russia, North America, and Oceania. Japan has an age-adjusted incidence rate of 36.6 cases per 100000 people and an age-adjusted mortality rate of 11.3 per 100000 people, both of which are high according to international standards.^3)^

Although the incidence of colorectal cancer has been increasing, PNS associated with colorectal cancer remains extremely rare, with published evidence limited to sporadic case reports. To our knowledge, no available epidemiological data regarding its incidence is available.^4–7)^ Furthermore, reports of colorectal cancer-associated PNS without detectable onconeural antibodies are even more uncommon.^8,9)^

Here, we report the case of a patient with suspected PNS based on his steroid-responsive impaired consciousness and the presence of sigmoid colon cancer; however, the patient tested negative for onconeural antibodies. The impaired consciousness did not recur even after reducing the steroid dose through tumor treatment, and the patient was comprehensively diagnosed with PNS.

## CASE PRESENTATION

**Fig. 1:**
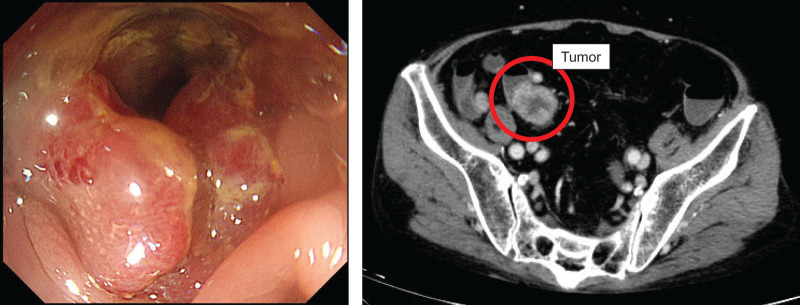
Sub-obstructive sigmoid colon cancer is observed. The clinical stage was cT3, N1a, M0, cStage IIIB (UICC TNM 8th).

A 79-year-old man was initially treated with 20 mg of prednisolone (PSL) at his previous hospital for recurrent arthritis and lower-extremity cellulitis (day 0). The patient was referred to our internal medicine department for further evaluation and treatment. Simultaneously, melena was noted at this previous hospital, and colonoscopy revealed sub-obstructive sigmoid colon cancer (**Fig. 1**), leading to a referral to the gastroenterological surgery department. He was initially diagnosed with gout and the PSL dose was gradually tapered. After tapering, a sigmoid colectomy was planned and a colonic stent was placed for the patient’s sub-obstructive sigmoid colon cancer classified as cT3N1aM0 and cStage IIIB (Union for International Cancer Control TNM 8th). Clinically, the patient had advanced sigmoid colon cancer; however, serum tumor markers, including carcinoembryonic antigen (CEA) and carbohydrate antigen 19-9 (CA19-9), showed only minor fluctuations during the clinical course and were not consistently elevated. Importantly, these changes did not correlate with disease progression, recurrence of impaired consciousness, or treatment interventions, including surgical resection and RFA. Consequently, serial measurements of CEA and CA19-9 were not informative for assessing the clinical course of the disease in this case.

Immediately after completion of PSL tapering on day 16, the patient’s arthritis and lower-extremity cellulitis recurred. Additionally, the patient developed impaired consciousness, initially presenting as somnolence, and worsening oxygenation. Various causes of impaired consciousness were investigated and excluded, including heart failure, encephalitis, stroke, pneumonia, and electrolyte abnormalities.

A head MRI scan revealed signs of old cerebral infarction, old cerebellar hemorrhage, chronic ischemic changes, and age-related cerebral atrophy; however, no lesions that could explain the current impaired consciousness were identified.

In addition, CSF examination revealed mildly elevated protein levels (45 mg/dL; normal range, 10–40 mg/dL), normal glucose levels (65 mg/dL; normal range, 50–75 mg/dL), and a normal cell count (1 cell/3 mm^3^). Furthermore, interleukin-6 was mildly elevated (5.6 pg/mL; normal range, 0–4 pg/mL). Taken together, these findings were consistent with inflammatory CSF changes without evidence of bacterial or viral infection.

Although the patient developed rapidly progressive impaired consciousness, no additional neurological features such as cerebellar ataxia, peripheral neuropathy, myoclonus, ocular motor disturbance, or gastrointestinal dysmotility were observed.

The rapidly progressive impaired consciousness in the presence of advanced sigmoid colon cancer raised the possibility that it was attributable to a PNS associated with sigmoid colon cancer.

However, the serum testing for paraneoplastic neurological syndrome-associated antibodies was performed using a commercially available panel (PNS12; BML, Tokyo, Japan), and all tested antibodies were negative (**Table 1**).

**Table 1:** Results of a commercial immunoblot assay for onconeural antibodies associated with paraneoplastic neurological syndromes (PNS12; BML, Tokyo, Japan)

Antibody	Day 28	Day 128
AMPH (amphiphysin)	–	–
CV2 (/CRMP5)	–	–
PNMA2 (Ma2/Ta)	–	–
Ri	–	–
Yo	–	–
Hu	–	–
recoverin	–	–
SOX1	–	–
titin	–	–
zic4	–	–
GAD65	–	–
Tr(DNER)	–	–

While an association between the patient’s impaired consciousness and PNS related to sigmoid colon cancer could not be completely excluded, the neurological presentation did not meet the criteria for either high-risk or intermediate-risk phenotypes according to the updated PNS diagnostic criteria.^10)^

According to the PNS-Care Score, the clinical phenotype was scored as 0 points because the neurological manifestations were limited to impaired consciousness without features of defined high- or intermediate-risk phenotypes. The laboratory level was scored as 0 points due to the absence of detectable onconeural antibodies, and the cancer component was scored as 4 points based on the presence of advanced sigmoid colon cancer.

Consequently, the PNS-Care Score was calculated as 4 points (clinical level 0, laboratory level 0, cancer 4), corresponding to a diagnosis of possible PNS.

Resumption of 40 mg of PSL on day 33 led to an improvement of the patient’s impaired consciousness. We determined that safe resection would be difficult with this PSL dose and postponed the planned sigmoid colectomy. Fortunately, stent placement improved the symptoms caused by the sigmoid colon cancer obstruction.

**Fig. 2:**
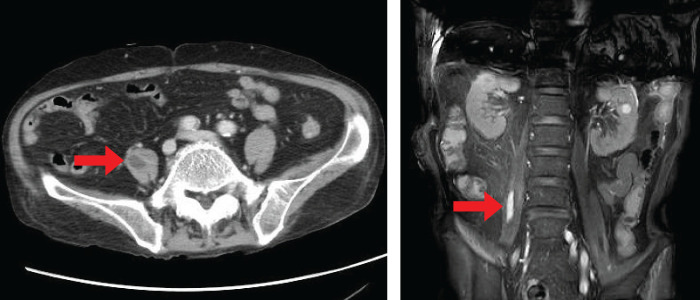
An iliopsoas abscess is detected in the right iliopsoas.

The PSL dose was gradually tapered to 20 mg on day 44, but impaired consciousness recurred. In addition, an increased inflammatory response was observed and imaging revealed an iliopsoas abscess (**Fig. 2**). Broad-spectrum antibiotic therapy with meropenem was initiated, and the PSL dose was increased to 40 mg due to the patient’s impaired consciousness. Disseminated intravascular coagulation (DIC) was noted during the course of the disease and *Klebsiella pneumoniae* was detected in blood cultures. Accordingly, the antibiotic was replaced with ceftizoxime sodium. The iliopsoas abscess subsequently shrank, and the patient’s impaired consciousness was stabilized with a dosage of 35 mg of PSL. Due to the trade-off between the risk of recurrence of impaired consciousness due to further reduction in the PSL dose and the risk of prolonged infection due to continued high-dose PSL, conservative treatment was deemed difficult, and sigmoid colectomy was recommended. The risk of anastomotic leakage is high during the administration of high-dose PSL. Given these considerations, a minimally invasive laparoscopic Hartmann’s procedure was performed.

**Fig. 3:**
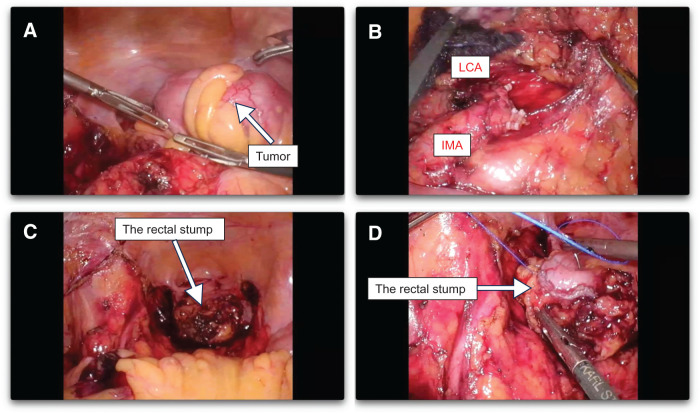
Intraoperative images of laparoscopic sigmoid resection. (**A**) The tumor was located in the sigmoid colon. (**B**) The inferior mesenteric artery (IMA) and the left colic artery (LCA) preserved low-ligation D3 lymphadenectomy was performed. (**C**) The anal side of the rectum was transected. (**D**) The rectal stump was closed with buried sutures.

**Fig. 4:**
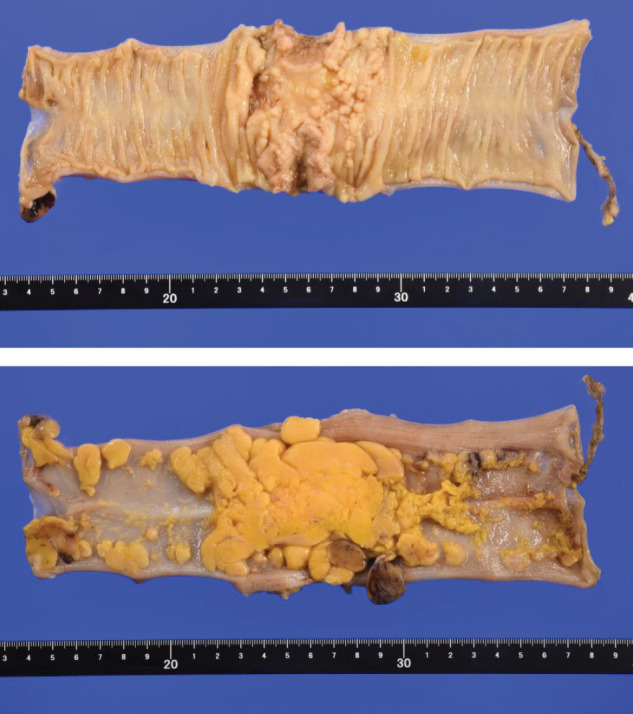
Sigmoid colon cancer: S, type2, 32 × 30 mm, 50/75 = 66.7%, tub2 > tub1, pT3, Ly1b, V1b, Pn1a, BD2, pPM0 (100 mm), pDM0 (100 mm), pRM0, pN0 (0/7), pM0 pStage IIA, R0, Cur A.

A total of five ports were inserted into the abdomen. The tumor was located in the Sigmoid colon (**Fig. 3A**), and the left colic artery preserved low-ligation D3 lymphadenectomy was performed (**Fig. 3B**). The anal side of the rectum was transected (**Fig. 3C**), and the stump was closed with buried sutures (**Fig. 3D**) and a colostomy was performed. The pathological diagnosis of the sigmoid colon cancer was pT3N0M0 and pStage IIA (**Fig. 4**). Histologically, the tumor was a moderate to well-differentiated adenocarcinoma (tub2 > tub1). No additional immunohistochemical staining was performed, as there were no pathological findings requiring further characterization beyond routine histological evaluation.

Because the patient was elderly and had a decline in activities of daily living due to prolonged treatment, postoperative systemic chemotherapy was not considered appropriate. Accordingly, genetic analyses, including RAS and BRAF mutation status and mismatch repair (MMR) status, which are primarily performed to guide systemic chemotherapy, were not conducted in this case.

During the perioperative period, red blood cell and platelet transfusions were required because of lingering DIC. Postoperatively, the DIC gradually improved and the PSL dose was gradually tapered. On POD 40 (day 115), the PSL dose was reduced to 2.5 mg.

**Fig. 5:**
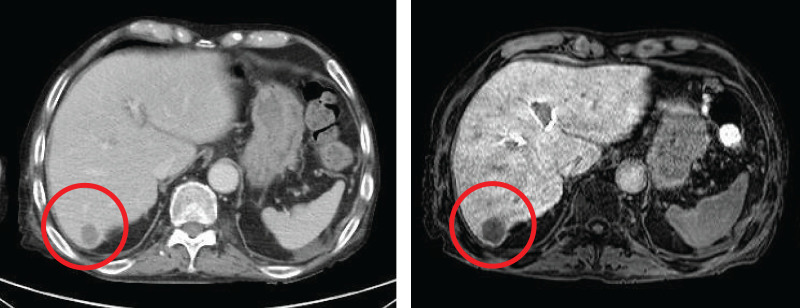
A micronodule detected in the right lobe of the liver indicated early liver metastasis.

**Fig. 6:**
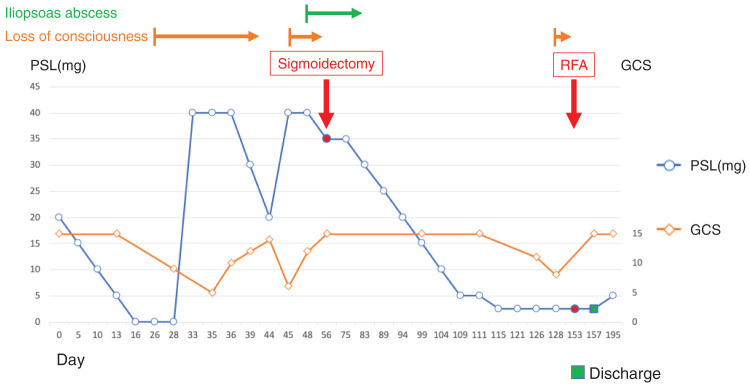
Steroid dose and event timeline. Day 0 was defined as the day of steroid treatment initiation. The Glasgow coma scale (GCS) was also included as an index of impaired consciousness. PSL, prednisolone; RFA, radiofrequency ablation

However, a new CT scan revealed a micronodule in the right lobe of the liver and antibiotic treatment was initiated because of a suspected liver abscess. During this period, the patient developed a lumbar compression fracture and his functional status deteriorated. Rehabilitation was continued; however, imaging tests showed that the liver nodule was growing, indicating early liver metastasis (**Fig. 5**). The patient again experienced impaired consciousness, and it was determined to be a PNS recurrence due to recurrent liver metastasis. Considering the patient’s decline in functional status, hepatic resection was deemed excessively invasive, and RFA was selected for treatment. The patient’s impaired consciousness improved after ablation and he was discharged on a PSL dose of 2.5 mg (**Fig. 6**).

Long-term steroid administration can cause iatrogenic adrenal insufficiency. Accordingly, the maintenance dose of PSL was eventually increased to 5 mg. Two years after surgery, the patient survived without recurrence.

## DISCUSSION

Here, we report the case of a patient with sigmoid colon cancer and impaired consciousness who was clinically diagnosed with autoantibody-negative PNS. Although steroid therapy temporarily improved the patient’s impaired consciousness, it recurred when the steroid dose was reduced and the increased steroid dose led to an iliopsoas abscess. As we faced a tradeoff between PNS treatment and infection control, the steroid dose was reduced by appropriately treating the tumor, namely, resection of the sigmoid colon cancer using the Hartmann procedure and RFA of the liver metastases.

Reports describing an association between colorectal cancer and PNS are not only limited but consist primarily of sporadic case reports.^4–7)^ Among these, colorectal cancer-associated PNS without detectable onconeural antibodies has been reported only exceptionally.^8,9)^

In the present case, the absence of onconeural antibodies allows only a diagnosis of “possible PNS” according to the latest diagnostic criteria.^10)^ Nevertheless, the complete resolution of steroid-dependent impaired consciousness following resection of the primary tumor strongly suggests a causal relationship between the neurological syndrome and the sigmoid colon cancer.

In this case, the diagnostic process for paraneoplastic neurological syndrome was conducted in a stepwise manner. First, common causes of impaired consciousness, including heart failure, infectious encephalitis, stroke, pneumonia, metabolic abnormalities, and electrolyte disturbances, were systematically excluded. Second, neuroimaging revealed no acute structural brain lesions that could explain the patient’s neurological symptoms, and CSF analysis demonstrated inflammatory but noninfectious findings. Third, although serum testing for onconeural antibodies was negative, the patient showed clear steroid responsiveness, suggesting an immune-mediated mechanism. Finally, the temporal association between tumor burden and neurological symptoms—namely, improvement after resection of the primary tumor, recurrence with the development of liver metastasis, and subsequent resolution after RFA—strongly supported a paraneoplastic etiology. Based on this sequential evaluation, the diagnosis of autoantibody-negative paraneoplastic neurological syndrome was considered most appropriate.

A particularly novel aspect of this case is that the neurological symptoms appeared to parallel not only the presence of the primary tumor but also the subsequent development and treatment of metastatic lesions. Specifically, impaired consciousness temporarily recurred when liver metastases occurred after primary tumor resection and subsequently resolved following RFA of the metastatic lesion. This clinical course suggests that paraneoplastic neurological features associated with the primary tumor may persist in metastatic lesions.

Furthermore, although impaired consciousness recurred after the appearance of liver metastasis, the severity of neurological symptoms was milder than that observed prior to the primary tumor resection. This may be attributable to the relatively small tumor burden, as the liver metastasis was solitary and limited in size. The absence of further neurological deterioration after RFA supports the possibility that neurological manifestations of PNS may, at least in part, correlate with overall tumor burden.

PNS is a diverse syndrome of neurological disorders associated with cancer, resulting from various immunological mechanisms. Symptoms arise from immune cross-reactions with host tissues owing to the secretion of functional hormones and peptides by tumors.^11,12)^ It is estimated that as many as 10%–15% of cancer patients suffer from PNS, which is the second leading cause of death in cancer patients after cancer itself.^13)^

Diagnostic criteria for PNS were proposed in 2004.^14)^ However, as time progresses, a deeper understanding of the clinical, immunological, and oncological diversity is needed, and diagnostic criteria were reevaluated in 2021.^10)^ These criteria replaced “classical syndromes” with “high-risk phenotypes” and proposed the introduction of new “intermediate-risk phenotypes.” Additionally, “onconeural antibodies” were replaced with “high-risk antibodies” (>70% associated with cancer) and “intermediate-risk antibodies” (30%–70% associated with cancer), depending on the strength of their association with cancer. A method has been proposed to classify the likelihood of PNS into three categories based on the PNS care score, which integrates the clinical phenotype, antibody type, presence or absence of cancer, and follow-up time.

In parallel, Graus et al. proposed a syndrome-oriented diagnostic approach for autoimmune encephalitis in 2016, which is also applicable to paraneoplastic encephalitis, emphasizing that the diagnosis can be established based on clinical presentation and supportive findings even in the absence of detectable autoantibodies.^15)^

However, while PNS is generally suspected when autoantibodies are detected, it is said that 30%–40% of PNS cases are autoantibody negative.^2)^ Diagnosis based on the presence of autoantibodies alone is difficult. Furthermore, the sensitivity of autoantibody immunodot assays varies depending on the antibody used,^16)^ indicating a high potential for false negatives.^17)^ In this case, the two autoantibody tests were negative, but it is possible that they were false negatives.

In this context, although the PNS care score did not reach the threshold for a definite or probable diagnosis, this scoring system is primarily intended for classification rather than exclusion. In antibody-negative cases with atypical neurological phenotypes, a syndrome-based diagnostic approach remains appropriate. Based on these findings, we diagnosed this case as seronegative paraneoplastic PNS, based on the clinical course, mildly inflammatory CSF findings, steroid responsiveness, and sustained neurological improvement after tumor treatment.

Steroid administration or direct tumor treatment is recommended when PNS is clinically suspected, even if direct tumor invasion or metastasis, nutritional disorders, metabolic disorders, coagulation disorders, side effects of chemotherapy or radiation therapy, or immunosuppression have been ruled out; other causes of impaired consciousness (e.g., heart failure, encephalitis, stroke, pneumonia, or electrolyte abnormalities) have been ruled out; and autoantibodies are negative. As immediate tumor removal during impaired consciousness is not practical, it seems appropriate to first confirm whether steroids improve impaired consciousness, as in this case.

Steroids exert potent immunosuppressive effects. A report comparing the “risk of infection” between steroid and non-steroid groups based on a pool of 71 randomized controlled trials found a 60% increased risk of infection in the steroid-treated group.^18)^ Simultaneously, no increased risk of infection has been reported in patients receiving a daily dose of less than 10 mg or a cumulative dose of less than 700 mg (PSL equivalent). However, in this case, the total PSL dose was 750 mg over 48 days that led to the onset of the iliopsoas abscess, reaching an average daily dose of 15.6 mg and posing a very high risk of infection.

Continued steroid administration was suspected to pose a risk of further worsening of the infection. Taking these risks into consideration, we performed a minimally invasive laparoscopic sigmoid colectomy. We also performed RFA for liver metastases, thereby achieving tumor control and weaning the patient from high-dose steroids.

When deciding to treat patients with steroids, careful attention should be paid to dosage, and alternative treatments other than steroids should always be considered.

Despite the clinical suspicion of PNS, definitive confirmation was challenging because all onconeural antibody test results were negative. This limitation underscores the need to consider PNS even in antibody-negative cases.

## CONCLUSIONS

PNS should always be considered the cause of neurological symptoms in patients with cancer. However, considering the possibility that autoantibodies may not be detected, including false negatives, in cancer patients in whom other causes of impaired consciousness are ruled out, it is important to suspect PNS and provide multidisciplinary treatment that appropriately combines treatment for the underlying tumor or steroid therapy, even if autoantibodies are negative.

## DECLARATIONS

### Availability of data and materials

The datasets generated and/or analyzed in the current study are not publicly available because of patient privacy concerns.
